# Increasing digitalization is associated with anxiety and depression: A Google Ngram analysis

**DOI:** 10.1371/journal.pone.0284091

**Published:** 2023-04-07

**Authors:** Gisbert Wilhelm Teepe, Edda Magareta Glase, Ulf-Dietrich Reips

**Affiliations:** 1 Department of Management, Economics and Technology, Centre for Digital Health Interventions, Eidgenössische Technische Hochschule (ETH) Zurich, Zurich, Switzerland; 2 Chair of Organizational Studies, University of Konstanz, Konstanz, Germany; 3 Department of Psychology, University of Konstanz, Konstanz, Germany; Regional Health Care and Social Agency of Lodi, ITALY

## Abstract

The prevalence of anxiety disorders and depression are rising worldwide. Studies investigating risk factors on a societal level leading to these rises are so far limited to social-economic status, social capital, and unemployment, while most such studies rely on self-reports to investigate these factors. Therefore, our study aims to evaluate the impact of an additional factor on a societal level, namely digitalization, by using a linguistic big data approach. We extend related work by using the Google Books Ngram Viewer (Google Ngram) to retrieve and adjust word frequencies from a large corpus of books (8 million books or 6 percent of all books ever published) and to subsequently investigate word changes in terms of anxiety disorders, depression, and digitalization. Our analyses comprise and compare data from six languages, British English, German, Spanish, Russian, French, and Italian. We also retrieved word frequencies for the control construct “religion”. Our results show an increase in word frequency for anxiety, depression, and digitalization over the last 50 years (r = .79 to .89, p < .001), a significant correlation between the frequency of anxiety and depression words (r = .98, p < .001), a significant correlation between the frequency of anxiety and digitalization words (r = .81, p < .001), and a significant correlation between the frequency of depression and anxiety words (r = .81, p < .001). For the control construct religion, we found no significant correlations for word frequency over the last 50 years and no significant correlation between the frequency of anxiety and depression words. Our results showed a negative correlation between the frequency of depression and religion words (r = -.25, p < .05). We also improved the method by excluding terms with double meanings detected by 73 independent native speakers. Implications for future research and professional and clinical implications of these findings are discussed.

## Introduction

Anxiety and depression are common health issues affecting hundreds of millions of people worldwide [1]. The already rising prevalence of mental health issues [2] is expected to rise further due to the ongoing COVID-19 pandemic [3]. This increase in prevalence is met by already thinly stretched resources for treating mental health [4].

Efforts to improve mental healthcare either focus on the improvement of face-to-face therapy [5], matching the correct treatment to the person [6], or the development of digital health interventions that are proposed to increase the reach or improve the effectiveness of treatments [7]. Other works try to identify underlying neurological functions or dysfunctions causing these illnesses to improve treatments [8]. These efforts have yielded a significant improvement in care over the last decades [5] and contributed to a better understanding of underlying dysfunctions. However, despite these improvements, systems struggle to sustain the steady influx of new cases, leading several to call for a stronger focus on preventing anxiety and depression [9].

To effectively improve the prevention of anxiety and depression, and to further advance their treatment, a promising approach could lie in identifying risk factors. Risk factors for anxiety and mood disorders on the individual level have been studied. Examples of such risk factors are major life events [10], chronic stress and acute stress [11], comorbidities with physical illnesses [12], loneliness [13,14], trauma [11], and childhood abuse [15]. Some risk factors, such as loneliness and alcohol misuse, have been hypothesized to be modifiable [16].

Studies investigating risk factors on a societal level have focused on social-economic status [17], social capital [18], unemployment [19], migration [20], and climate change [21]. The World Health Organization (WHO) summarizes these findings by stating that “*mental health and many common mental disorders are shaped to a great extent by the social*, *economic*, *and physical environments in which people live*” [17, p. 8]. Compton and colleagues [22] argue that these social determinants for mental health, exemplified in their work by income inequality and poor education, need to be addressed by public policies and social norms.

Different works have investigated risk factors on the individual level associated with or accelerated by ongoing digitalization. While the scientific management and information system community seems to be highlighting the positive effects of digitalization on the economy and innovation, work from the clinical and social sciences also highlights some potential negative effects. Digitalization may potentially be associated with high levels of uncertainty as it is unclear what is to be expected after the digitalization-related changes take place and whether those changes will influence the individual’s employment or private life. These uncertainties could, in turn, lead to increased anxiety, a feeling of worry, or discomfort.

A digital source of uncertainty and worries is the use of social media. Several studies investigated the impact of social media and social media use on mental health. Different works have investigated risk factors on the individual level, with results indicating that not the quantity of use but the purpose for using social media is relevant [14,23], and some studies even show no clear evidence for a problematic relationship between social media and mental health [24]. The authors explained this finding about general skepticism toward social media with the fact that new forms of media have always sparked the worry of being dangerous.

Furthermore, *technostress*, or the experience of stress due to an inability to adapt to new technologies [25], manifests in various conditions such as job dissatisfaction [26], perceptions of being drained by the use of technological devices [27], feelings of exhaustion, loss of motivation, frustration, and burnout [28]. Further problems associated with digitalization, such as replacement fear [29], stressful hyper-connectivity [30], an increase in a cognitively demanding and simultaneous decrease in manual jobs [31], and digitalization anxiety [32], may further cause or aggravate mental health problems.

Other studies reported fear and other negative outcomes due to digitalization. Kim and colleagues [33] and Turel and colleagues [34] found that exposure to digitalization in the work environment can exhaust employees to the point of poor mental health, sometimes observed as job burnout. Ferguson and colleagues [35] reported a negative spillover effect of unpleasant technology-related constant connectivity on family or other live domains, causing work-family conflicts. Furthermore, on an emotional level, Tarafdar and colleagues [36] reported that technology use evoked negative attitudes and emotions.

However, evidence investigating the association between ongoing digitalization and mental health is rare, specifically before the COVID-19 pandemic. In a Scopus search using the search term (*“Digitalization” OR “Digitalisation”*) *AND “Mental Health”*, we found 86 publications (out of 890 screened publications, date of search 1^st^ of June 2022) that had some connection to digitalization or mental health but not one examined the connection between ongoing digitalization and anxiety or depression on a broader level. While these changes associated with digitalization may potentially affect large numbers of individuals in different situations, the methods used to investigate the influence of these changes remained the same. Researchers rely on longitudinal studies, survey self-reports, questionnaires [37], and interviews [38]. So far, few studies have leveraged the vast and various data available (i.e., big data) to investigate the connection between digitalization and anxiety and depression. This gap in research is surprising because there is, for example, other work highlighting that digitalization may lead to higher unemployment, especially in populations with a lower social-economic status that has been shown to be additionally affected more strongly by mental health problems [39].

To fill the gap, we aim to use the vast amount of data available from Google Books Ngram Viewer (henceforth: Google Ngram) to investigate the impact of digitalization on anxiety and depression by reviewing the historically disjointed works of literature on anxiety and depression and digitalization. Google Ngram is a large corpus of books (more than 8 million books or more than 6 percent of all books ever published) that has been used in related linguistic big data approaches to investigate societal trends and changes [e.g., 40,41]. While online self-report data used in other studies may be hampered by methodological issues and, for example, privacy concerns [42], Google Ngram relies on data from published scientific and fiction (fiction is only available for the English corpus) books and can thus be considered a less biased source in that sense. By targeting the effect that digitalization may have on anxiety and depression and using this novel approach, we aim to advance the social science branch in the following ways.

First, in line with its surge in public attention, we investigate the relative frequency of anxiety, depression, and digitalization terms over the last 50 years. We hypothesize that the relative frequency of each of these terms will increase over the years covered. Second, we aim to provide further evidence for the co-occurrence of depression and anxiety. Due to the often-reported comorbidity of both mental health diseases [43], we expect these word lists to be strongly associated. Third, we aim to investigate the ongoing digitalization’s impact on anxiety and depression. Existing research on this influence has focused on technostress [e.g., 44], challenging hyper-connectivity [30], and digitalization anxiety [32]. These findings indicate a possible link between digitalization and increasing anxiety and depression rates. We argue that these mental disorders are multidimensional and therefore propose that they need to be investigated using various approaches. Here we present and analyze data from Google Ngram. By investigating the possible link between anxiety, depression, and digitalization, we aim to contribute to a broader understanding of how societal changes may influence these mental health disorders. We hypothesize that word lists semantically describing these disorders are strongly associated with one another. Last, we aim to advance methodological approaches to investigate the prevalence and causes of mental disorders by refining existing linguistic big data approaches [41], thereby contributing to developing complementary approaches to self-reports in clinical and social psychology research. We argue that the frequency of words in both scientific research, fictional literature, and news could be a relevant measure because research is driven by addressing pressing real-world problems, literature is influenced by Zeitgeist, and news reports address the most demanding societal issues.

## Materials and methods

We aim to investigate the general usage of word frequencies for anxiety, depression, and digitalization over the last 50 years via the Google Ngram database. Google Ngram is a digital repository for analyzing change through changes in relative word frequencies over a specific period available online [45]. It offers a big data perspective and allows for studying changes in various topics [46]. To this aim, we extracted anxiety and depression word lists from eight successive versions of the ICD-8 (International Classification of Diseases) to ICD-11 [47–49]. By using the ICD, we set as a criterion the widely applied international standard classification for diagnoses regarding mental and physiological health. The ICD classification allowed us to interpret and compare different health symptoms over a specific period. The digitalization word lists were derived from Brewster and Murray-Smith [50] and Wolf and Bartelheimer [51], covering the last 50 years. Following the procedure by Younes and Reips [41], we generated a set of 26 words most representative of the concepts of anxiety and depression and a set of 33 most representative words describing digitalization resulting in two separate lists (S1 and S2 Tables in S1 File).

Following the guidelines by Younes and Reips [41] (Procedure IV), we searched for synonyms using the standard online dictionary Roget’s Thesaurus (standard reference for English). We included all synonyms labeled “*most relevant*”, excluded all synonyms with a broader semantic meaning (e.g., “*interest*”, “*platform*”), and only focused on 1-grams because “*particular two-word combinations would be too infrequent in the corpus to show change over time*” [52, p. 1724]. This process yielded 85 words translated into five languages (German, Spanish, Russian, French, and Italian) and cross-validated by native speakers who are also proficient in English. We chose these six languages (British English, German, Spanish, Russian, French, and Italian) as they are the only languages spoken on the European continent available on Google Ngram. In the subsequent step, 73 native speakers translated the word lists by themselves without pre-existing translation using independent back-translation [53,54] to optimize the translatability. We merged all translations, excluded terms with double meanings detected by native speakers, and generated two final sets of 59 words that exist in all six languages. These lists are displayed in the (S1 and S2 Tables in S1 File).

We analyzed word inflections to quantify the final word lists using the tag “_INF” for each word. This method finds and compares inflection frequencies for words (Younes and Reips [41]). This consistency check confirmed that our originally selected word lists displayed the highest frequency. Additionally, we double-checked the word lists for correctness. Following Lin and colleagues [55], we compared the most frequent word in each corpus with the most frequent noun in each language to account for the influx of data (British English = “*time*”,”*people*”; German = “*Zeit*“, “*Jahre*“; Spanish =“*parte*”, “*años*“; Russian = “*эго*“; *“он“*; French = “*temps*”,”*partie*”; Italian =“*parte*”,”*tempo*”, p. 171). Lastly, we calculated summed z-scores for each language and each word list.

Additionally, we compared the generated word lists with a control list, namely the religion word list [41,56]. In doing so, we followed the approach by Younes and Reips [41] and were able to control for a general misleading trend counterfactually.

We used the final set of words as the Google Ngram search terms. We collected data on the word frequency per year in percent. Google Ngram divides the number of a word’s yearly appearances by the total number of words in the corpus in that year and generates charts of those word frequencies [41], resulting in a longitudinal analysis over a period. We extracted the Google Ngram results using the Git Hub repository econpy/google-ngrams available under (https://github.com/econpy/google-ngrams, MIT Licence). We retrieved all available data up to 2019, the latest year the Google Ngram data is available. Thus, our investigation only considers the time before the ongoing COVID-19 pandemic and for languages spoken on the European continent. Our study investigates changes over the 50 years before 2020 in Europe.

## Results

**Fig 1:**
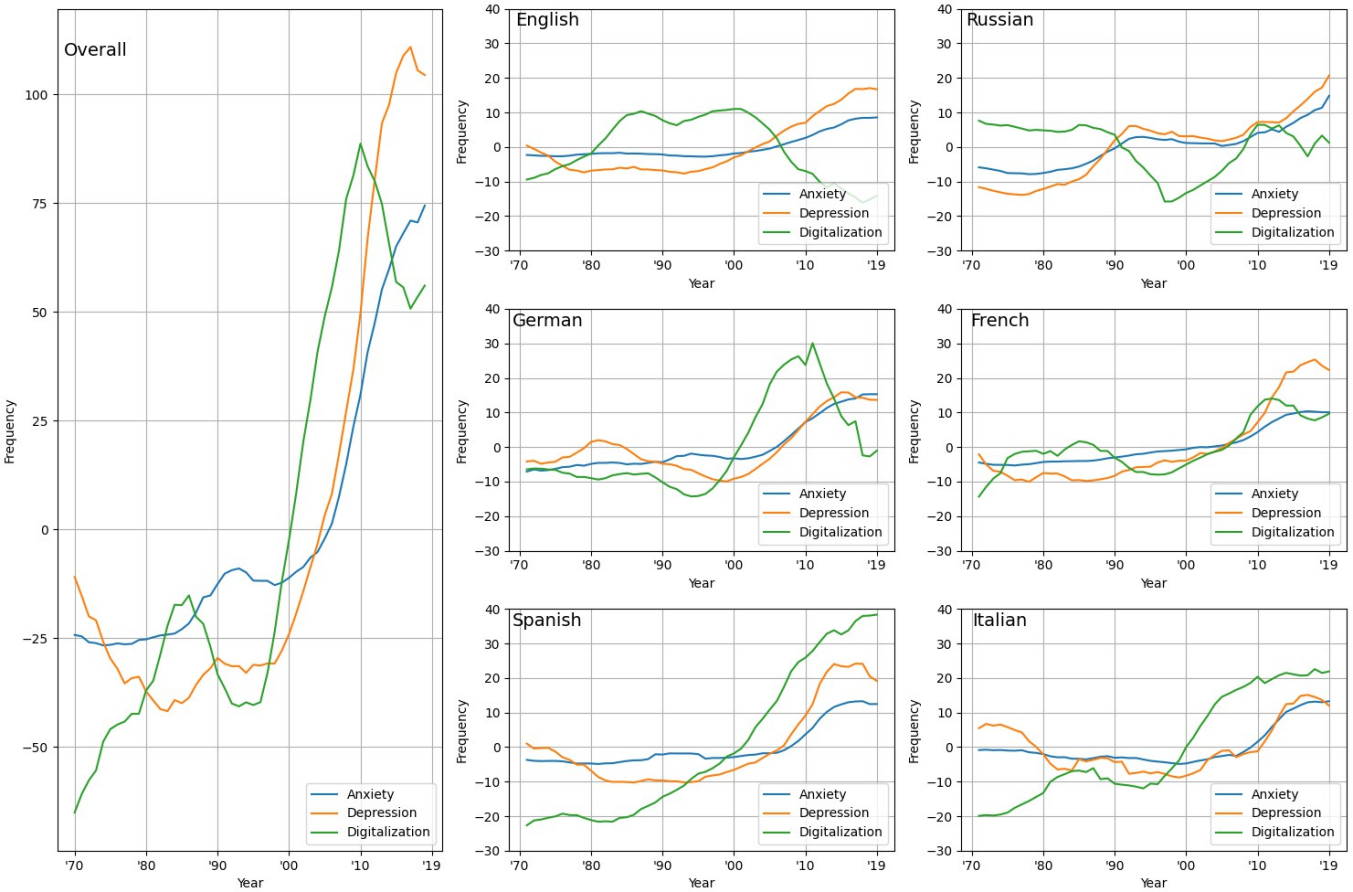
List-averaged word frequencies from 1970 until 2019 for each category and language. Frequencies (in percent) are z-transformed, most common word controlled, and summed over all words.

We calculated correlation coefficients between the word frequencies in percentage and years. By doing so, we assumed that the more frequent a word was, the larger its proportional influence was per year. Changes in frequencies from 1970 until 2019 over all languages and for each language can be found in Fig 1.

Separate correlations were calculated for each language and the overall combined frequency scores of terms from all languages. Table 1 illustrates the result of the analysis over all languages, and the results for each language can be found in S4–S10 Tables in S1 File.

**Table 1:** Correlation matrix over all languages for mean frequency of word groups (z-transformed) from 1970 until 2019.

		1	2	3	4	5
	**1 Anxiety**					
	**2 Depression**	.87***				
	**3 Digitalization**	.81***	.81***			
	**4 Years**	.87***	.79***	.89***		
**Control**						
	**5 Religion**	-.18	-.25*	-.01	.22	

Note.

* p < .05;

** p < .01;

*** p < .001.

### Anxiety

We found a strong positive significant correlation between anxiety and years over all languages (r = .87, p < .001) and for each individual language: British English (r = .81, p < .001), German (r = .88, p < .001), Spanish (r = .82, p < .001), Russian (r = .93, p < .001), French (r = .93, p < .001), and Italian (r = .62, p < .001).

### Depression

We found a strong positive significant correlation between depression and years over all languages (r = .94, p < .001) and positive correlations of varying strength for the individual languages: British English (r = .8, p < .01), German (r = .6, p < .001), Spanish (r = .7, p < .001), Russian (r = .93, p < .001), French (r = .84, p < .001), and Italian (r = .32, p < .01).

### Digitalization

We found a positive significant correlation between digitalization and years over all languages (r = .89, p < .001) and significant correlations for each language. The correlations were moderately to strongly positive for German (r = .65, p < .001), Spanish (r = .95, p < .001), French (r = .72, p < .001), and Italian (r = .94, p < .001), but slightly negative for British English (r = -.29, p < .01) and Russian (r = -.33, p < .01).

### Anxiety and depression

We found a strong positive significant correlation between anxiety and depression over all languages (r = .98, p < .001) and significant and strongly positive correlations for each of the languages: British English (r = .96, p < .001), German (r = .88, p < .001), Spanish (r = .94, p < .001), Russian (r = .99, p < .01), French (r = .98, p < .001), and Italian (r = .9, p < .001).

### Anxiety and digitalization

We found a strong significant correlation between anxiety and digitalization over all languages (r = .81, p<0.001) and strong positive significant correlations for German (r = .56, p < .001), Spanish (r = .91, p < .001), French (r = .79, p < .001), and Italian (r = .66, p < .001). We also found strong negative correlations for British English (r = -.73, p < .001) and Russian (r = -.34, p < .01).

### Depression and digitalization

We found a positive significant correlation between depression and digitalization (r = .81, p<0.001) and strong positive significant correlations for German (r = .51, p < .001), Spanish (r = .87, p < .001), French (r = .75, p < .001), and Italian (r = .43, p < .001). We also found strong negative correlations for British English (r = -77., p < .001) and Russian (r = -.39, p < .001).

## Control analyses

First, to validate our results, we conducted further control analyses. Following the suggestions by Younes and Reips [41], we additionally used the Google Ngram English fiction corpus to test the robustness of our findings. Fiction books are less influenced by trends in scientific findings and can serve as a more general representation of a broad trend [57]. Because the fiction corpus is only available in English, we could only correlate word frequencies for English fictional books. The significant overall trend could be confirmed for anxiety and depression (r = .64, p < .001) and for depression and digitalization (r = .71, p < .001), while no significant result for anxiety and digitalization was observed (r = .16, p = .28) (all correlations can be seen in S14 Fig in S1 File). The results from the English fictional corpus thus partly validate the English word lists.

Second, to further validate our final word list for anxiety, depression, and digitalization, we used another word list, namely the *religion word list* [41,56]. This additional word list served as a control to test the robustness of our findings. The religion word list from Younes and Reips [41] contains, for example, words like “*God*”, “*Angel*”, “*Clergy*,” and “*Prayer*”. By using this established list, we maintain comparability between previous work, our work, and -hopefully—future work.

Third, following the procedure by Younes and Reips [41], we used (I) all European language corpora available on Google Ngram, (II) crossed-checked with English Fiction corpora, (III) controlled for word inflections using the tag “_INF”, (IV) controlled for synonyms using the standard online dictionary Roget’s Thesaurus, (V) z-standardized, and (VI) validated the wordlists by accounting for the most common nouns [55] in each language. In an additional step, native speakers translated the word lists without pre-existing translation using independent back-translation again [53,54]. This process yielded a total of 19 control words. Thus, we validated our study lists and found that the correlation between the religion word list and anxiety (r = -.18, n.s.), depression (r = -.25, n.s.), and digitalization (r = -.01, n.s.) is negative and not significant. In an additional step, we included the religion control list to validate the correlation between our primary word lists. We used the religion word list as a control list because there is a thematic overlap in the content of religion and anxiety/depression. A systematic review by Braam and Koenig [58] indicated a correlation between depression and religion. The final set of our control list is displayed in S3 Table in S1 File.

**Fig 2:**
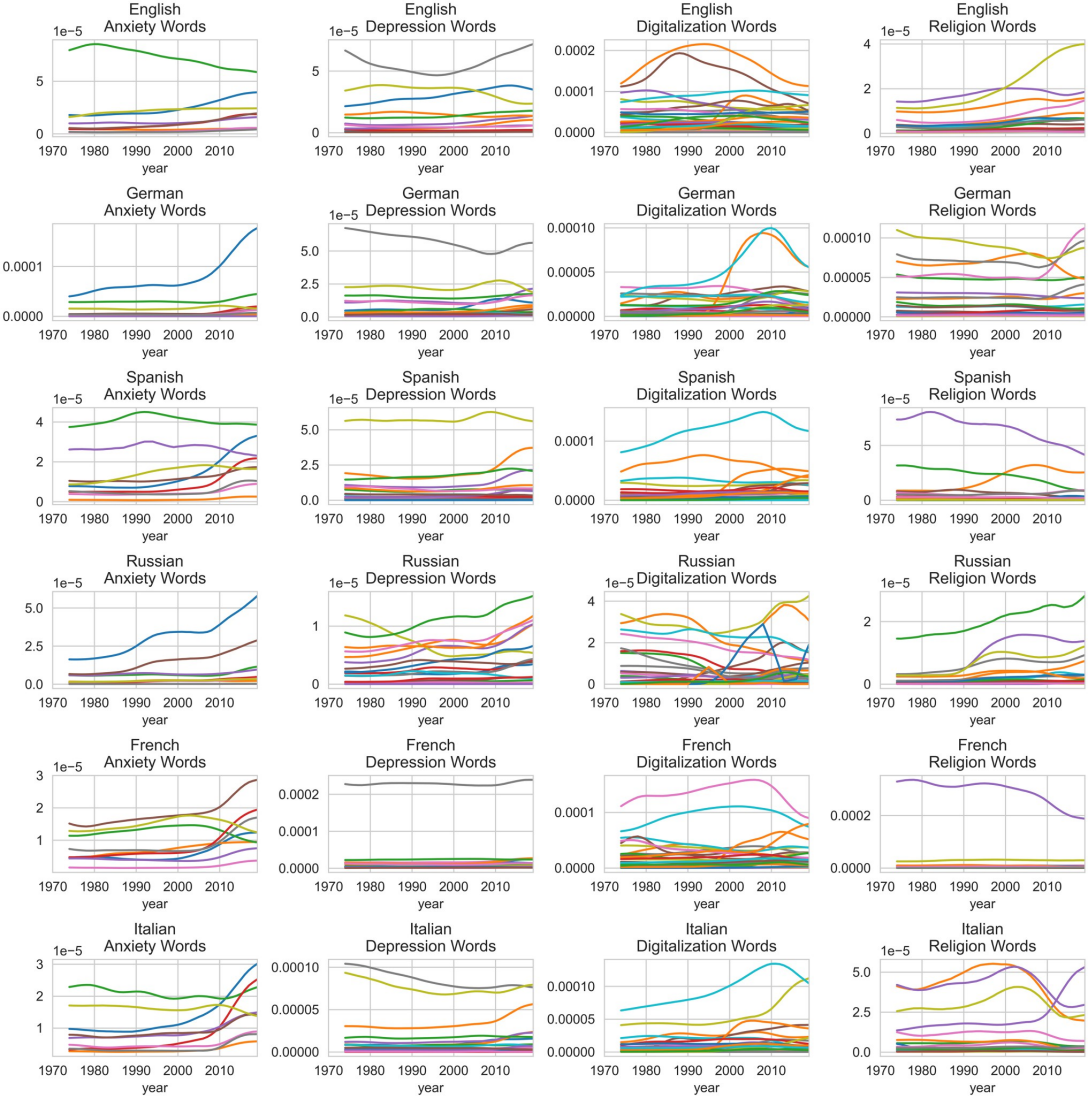
Rolling mean word frequencies from 1970 until 2019 for each word by each category and language, considering five years.

Last, our subsequent analysis will address a potentially confounding factor underlying our findings: a general trend of the increasing (scientific) literature addressing depression, anxiety, and digitalization. Trends and seasonality (i.e., non-stationary data) are problematic for economic forecasting [59]. However, our work does not aim to predict future changes but to investigate a historical trend over the years. To account for the potentially strong impact of certain words, we applied the procedure of z-transforming, adjusting each word using the most common word and averaging over all words by Younes and Reips [41] described above. Regardless, in Fig 2, we plotted the rolling mean for each word, considering five consecutive years to investigate whether we can observe a general upwards trend indicating non-stationarity.

We observed no increase in mean changes for English anxiety words, English depression words (except for one word), English digitalization words (except for two words), German anxiety words (except for one word with a substantial increase of mean changes), German depression words (except for one word), German digitalization words (except for two words), Spanish depression words (except for one word with an increase in the 2010s), French depression words, French digitalization words, and French religion words (except for one word with a significant decrease). We observed an increase in mean changes for English religion words (minor changes), Russian depression words, Russian religion words, French anxiety words, French anxiety words, and Italian anxiety words. We observed a decrease in mean changes for Spanish religion words. We observed mixed changes for German religion words, Spanish anxiety words, Spanish digitalization words, Russian anxiety words (two words with consistently increasing mean changes), Russian digitalization words, Italian depression words, Italian digitalization words, and Italian religion words.

While some words in some languages have increased in rolling mean frequency, non-stationarity cannot be observed consistently over specific word lists. We did not observe a strong impact on specific word groups. A systematic evaluation that addresses non-stationarity approaches (e.g., differencing, log transformation, or power transformations) should be investigated in future work.

## Discussion

With this work, we aimed to investigate the association between ongoing digitalization and the growing prevalence of anxiety and depression. Due to the lack of studies investigating a potential relationship to digitalization at the broader level, we used and extended (independent forward and backward translation [53,54]), independent translators, and further languages) a linguistic big data approach using word frequencies derived from the Google Ngram Viewer.

We found strong positive correlations of word frequencies over all languages between anxiety and years (r = .87, p < .001), depression and years (r = .79, p < .001), and digitalization and years (r = .89, p < .001), but no significant correlation between religion and years (r = .22, p = .12; see visual representations in S1-S4 Figs in S1 File). Similar associations were found for anxiety and depression words and a negative correlation for religion when using only German words, only Spanish words, only French words, or only Italian words. English and Russian words showed slightly different results for anxiety and depression (English: r = .67, p < .001; Russian: r = .81, p < .0001), but for digitalization (English: r = -.73, p < .001; Russian: r = -.34, p < .0001) and religion the results were negative and significant. These findings largely support our hypothesis that anxiety, depression, and digitalization frequency jointly increased over the last 50 years compared to the control construct religion. The frequently described co-occurrence of anxiety and depression and our hypothesized strong association between anxiety and depression terms were supported by our results as well. These word groups’ frequency was strongly associated over all languages (r = .98, p < .001). Correspondingly, we observed similar associations between the frequencies of the word groups for all languages, including English fiction (r = .64 to .99; see S5-S13 Figs in S1 File).

A potential explanation for this trend is that new technologies have caused fear in individuals whenever they have been introduced. Mokyr and colleagues [60] argue that fear due to automatization has existed since the beginning of the industrial revolution. Employees feared and fear that they could lose their jobs due to societal change. Despite this fear, many did not lose their jobs. The quality of jobs tended to decline, however. Similar fear has been described in work focussing on the more recent impact of digitalization on job security and quality [60]. Further evidence for a relationship between digitalization and anxiety or depression may be represented in the rising prevalence of psychopathological problems such as computer anxiety [61], replacement fear, digitalization fear, internet anxiety, and technostress.

In addition to these emotional and psychopathological effects, our findings also highlight problems in the ongoing trend of treating anxiety and depression with digital solutions. Due to increasing demand, reduced (public) healthcare funding, changing regulations, and increased investment in digital health, researchers and start-ups have developed and investigated digital interventions for anxiety and depression over the last decades [62]. These digital interventions may involve no healthcare professional (i.e., standalone), combine face-to-face therapy with digital treatment (i.e., blended treatment, for further information, see [63]), or provide additional tools besides the standard of care (i.e., add-on). Digital interventions often use chatbots [64], modules derived from cognitive behavioral therapy [65], gamification [66], and monitoring of symptoms [67,68]. In some countries, they are already part of the health care services. In Germany, for example, standalone digital health interventions (DiGA) can be prescribed by a board-certified physician or psychotherapist, and insurers need to reimburse the costs for the digital intervention [62]. Due to the rising numbers and stretched resources, healthcare systems and patients need these new types of therapy to provide or receive adequate care [69]. However, further work needs to investigate for which individuals such a standalone digital intervention could be helpful and for which individuals’ digital therapies may aggravate their mental health problems.

In all of this, using Google Ngram may help review historical trends; researchers can gain insight into how mental health attitudes and understanding may have changed over time. This understanding may help in refining theories. Our results show that anxiety, depression, and digitalization correlate, supporting other findings outlined in the introduction. Regarding policy, our results may help to inform the development of more effective and evidence-based policies and programs for mental health by highlighting the importance of digitalization and its effects. In shaping policies, it is undoubtedly vital to see how language describing mental health symptoms and conditions tends to change over time. With additional future work, mental health professionals may be able to diagnose patients using words outside of the standard vocabulary to describe depression or anxiety. This may be especially useful when patients are unwilling or unable to report their symptoms explicitly.

With this study, we also aimed to further refine already existing linguistic big data approaches using Google Ngram. Using the guidelines Younes and Reips Feld [41] developed, we have extended the recommended methodology to ensure greater validity. First, 73 native speakers translated the word lists in two consecutive steps. By having independent native speakers involved, we can ensure that we have controlled for linguistic nuances, especially in verbs describing emotions. Furthermore, we can rule out *false friends* (e.g., German: sensibel, English: sensitive, false friend: sensible = vernünftig *OR* German: resignieren, English: give up, false friend: resign = zurücktreten), which most likely can only be detected by native speakers. Via our translators, we could also control for Zeitgeist and trends in language use. Using native speakers of different ages, we accounted for time- and age-portraying vocabulary use.

Second, we used independent back-translation. Independent back-translation allows for an extra level of proofing without the potential biases of a native speaker. This method is used, for example, in the translation of validated psychological questionnaires, but so far, to the best of our knowledge, it has not been combined with the Ngram methodology. Finally, we validated the English wordlists with the English fiction corpus. The English fiction corpus can serve as a more general representation of a broad trend [57]. We also referred to the classification system ICD [47–49]. We used the latest four versions to not only include the latest version of the classification system. With this procedure, we wanted to avoid a possible bias in framing the symptoms of anxiety disorder and depression.

## Limitations and directions for future research

The strengths of this study are the large corpus of data used, the further refined and extended rigorous method by Younes and Reips [41], and the use of control word lists. The study has some limitations, however. First, investigating the correlation between different word lists only provides further evidence for the proposed relationship between anxiety, depression, and digitalization. A causal effect of digitalization on anxiety and depression cannot be inferred even though a control construct (religion) was used. This is a common problem in related machine-learning approaches aiming to find digital (bio)markers for anxiety or depression. In these studies, a correlation is investigated between a digital outcome (e.g., phone calls made) and a symptom (e.g., social withdrawal). Causality between digital (bio)markers and the symptoms has yet to be demonstrated. One approach to address this shortcoming is to use self-reported and digital data to infer a construct to review whether the (bio)marker can be seen as a compliment or alternative to existing instruments [70]. In our work, the confirmation and relative specificity of the strong correlation between anxiety/depression and digitalization was controlled by using the construct of religion. We also used related work [e.g., 60] describing the effect of ongoing digitalization on anxiety and depression to provide further evidence for a potential causal relationship.

Also, as outlined in 2012 already by Snijders and colleagues [71], the problem of using big data to understand the underlying empirical micro-processes leading to the emergence of large-scale changes remains. We agree with Snijders and colleagues’ (2012) [71] conclusion that observed relations in big data have to be supported by survey data, which offers richer insights into the observed relationship. Regarding our analysis, this implies that we need to validate our found relations between digitalization, anxiety, and depression with survey data. As a primary indication of this connection, we refer to various findings in the introduction showing a relationship using survey results.

Furthermore, with the Ngram approach, we have focused on six languages: British English, German, Spanish, Russian, French, and Italian, to represent the European continent. In addition to the languages listed, simple Chinese and Hebrew are also available on the Google Ngram Corpus. Thus, we encourage future researchers to include these languages in replication analyses to be able to generalize beyond the European continent.

Additionally, specific to the Ngram approach, we rely on data derived from a source that uses scientific publications, books, and other outlets. Therefore, the database grows more by each year, and one could thus argue that what we find in it may be influenced by the general increase in publications. We aimed to address this increase in publications across all areas by using our approach of using the relative frequency adjusted for each year.

Finally, further research could investigate the impact of the COVID-19 pandemic (pre versus post) as done by Younes and Reips [72], where changes in religious terms were compared before and after the second world war. We would expect an increase in anxiety and depression, and digitalization due to mandatory lockdowns and substantial time working from home, as well as fears regarding a slowing economy, short-time work, and using the pandemic as an excuse for already planned downsizing. However, because the pandemic is ongoing, this analysis needs to be carried out at a later point.

Our results still need to be validated using other data (e.g., using data from the national surveys of the United States Center of Disease Control, using languages outside the European continent, and for other mental illnesses with varying prevalence. Future work could also investigate how certain events, such as COVID-19 and the Russian invasion of Ukraine, impact anxiety, depression, and digitalization. However, this data has not yet been released by Google Ngram (last checked February 2023).

## Conclusion

Our study analyzes the impact of digitalization on anxiety and depression by applying a linguistic big-data approach. Our research constitutes a new conceptualization to investigate the impact of digitalization on anxiety and depression and advances the method of using Google Ngram to gather insights regarding epidemiological questions. Also, we discuss related work describing the impact of digitalization on anxiety and depression (e.g., internet anxiety, technostress). For future research, we highlight the potential of this method’s potential to investigate the impact of historical events (Russian invasion of Ukraine, COVID-19 pandemic) and environmental changes (e.g., climate change) on anxiety and depression. Our results showed that measuring societal changes may be possible using a big linguistic data approach.

## Supporting information

S1 File Supporting information—Contains all supporting tables and figures. (DOCX)
