# Supplementary material for: Increasing digitalization is associated with anxiety and depression: A Google Ngram analysis
Source: PLoS One. 2023 Apr 7;18(4):e0284091. doi: 10.1371/journal.pone.0284091 (PMC10081798; doi:10.1371/journal.pone.0284091)
Supplement: S1 File — (DOCX) [file pone.0284091.s001.docx]

**Supporting Information**

**S1 Table. Finalized word set for Anxiety and Depression.**

|  | **British English** | **German** | **Spanish** | **Russian** | **French** | **Italian** |
| --- | --- | --- | --- | --- | --- | --- |
| **1** | Anxiety | Angst | Ansiedad | Страх | Anxiété | Ansia |
| **2** | Apprehension | Befürchtung | Aprensión | Опасение | Appréhension | Apprensione |
| **3** | Concern | Sorge | Preocupación | Волнение | Préoccupation | Preoccupazione |
| **4** | Depression | Depression | Depression | Депрессия | Dépression | Depressione |
| **5** | Sadness | Traurigkeit | Tristeza | Печаль | Tristesse | Tristezza |
| **6** | Melancholia | Melancholie | Melancolía | Меланхолия | Mélancolie | Malinconia |
| **7** | Panic | Panik | Pánico | Паника | Panique | Panico |
| **8** | Alarm | Alarmiertheit | Vigilancia | Тревога | Alarme | Allarme |
| **9** | Consternation | Bestürzung | Consternación | Смятение | Consternation | Costernazione |
| **10** | Horror | Entsetzen | Horror | Ужас | Horreur | Orrore |
| **11** | Despair | Verzweiflung | Desesperación | Отчаяние | Désespoir | Disperazione |
| **12** | Scare | Schreck | Susto | Испуг | Effrayer | Spavento |
| **13** | Nervousness | Nervosität | Nerviosismo | Нервозность | Nervosité | Nervosismo |
| **14** | Disquiet | Unruhe | Desasosiego | Нервность | Inquiétude | Turbamento |
| **15** | Impatience | Ungeduld | Impaciencia | Нетерпеливость | Impatience | Impazienza |
| **16** | Uncertainty | Unsicherheit | Incertidumbre | Неуверенность | Incertitude | Incertezza |
| **17** | Doubt | Zweifel | Duda | Смущение | Doute | Dubbio |
| **18** | Burden | Belastung | Carga | Нагрузка | Fardeau | Peso |
| **19** | Passivity | Passivität | Pasividad | Пассивность | Passivité | Passività |
| **20** | Laziness | Faulheit | Vaguería | Лень | Paresse | Pigrizia |
| **21** | Fatigue | Erschöpfung | Fatiga | Усталость | Fatigue | Fatica |
| **22** | Weakness | Schwäche | Debilidad | Слабость | Faiblesse | Debolezza |
| **23** | Apathy | Apathie | Apatía | Апатия | Apathie | Apatía |
| **24** | Irritation | Gereiztheit | Irritación | Раздражённость | Irrité | Irritato |
| **25** | Disinterest | Desinteresse | Desinterés | Равнодушие | Désintérêt | Disinteresse |
| **26** | Agitation | Unruhe | Agitación | Беспокойство | Agitation | Agitazione |

*Note.* Anxiety and depression word list based on ICD - 8 till ICD – 11 [63-65] and modified by the authors of this study based on the judgements of native speakers.**S2 Table. Finalized word set for Digitalization.**

|  | **British English** | **German** | **Spanish** | **Russian** | **French** | **Italian** |
| --- | --- | --- | --- | --- | --- | --- |
| **1** | Digitalization | Digitalisierung | Digitalización | Oцифровка | Digitalization | Digitalizzazione |
| **2** | Technology | Technologie | Tecnología | Технология | Technologie | Tecnologia |
| **3** | Automation | Automatisierung | Automatización | Автоматизация | Automatisation | Automazione |
| **4** | Mechanics | Mechanik | Mecánica | Механика | Mécanique | Macchinario |
| **5** | Robotics | Robotik | Robótica | Робототехника | Robotique | Robotica |
| **6** | Computer | Computer | Ordenador | Компьютер | Ordinateur | Computer |
| **7** | Template | Vorlage | Plantilla | Образец | Modèle | Template |
| **8** | Device | Gerät | Dispositivo | Прибор | Dispositif | Dispositivo |
| **9** | Machine | Maschine | Máquina | Машина | Machine | Macchina |
| **10** | Apparatus | Apparat | Aparato | Аппарат | Appareil | Apparato |
| **11** | Gadget | Gadget | Gadget | Гаджет | Gadget | Gadget |
| **12** | Internet | Internet | Internet | Интернет | Internet | Internet |
| **13** | Automatic | Automatisch | Automático | Автоматический | Automatique | Automatico |
| **14** | Automated | Automatisiert | Automatizado | Автоматизированный | Automatisé | Automatizzato |
| **15** | Electric | Elektrisch | Eléctrico | Электрический | Electrique | Elettrico |
| **16** | Electronic | Elektronisch | Electrónico | Электронный | Électronique | Electrónica |
| **17** | Mechanical | Mechanisch | Mecánico | Механический | Mécanique | Meccanico |
| **18** | Server | Server | Servidor | Сервер | Serveur | Server |
| **19** | Chat | Chat | Chat | Чат | Discuter | Chat |
| **20** | Communication | Kommunikation | Comunicación | Коммуникация | Communication | Comunicazione |
| **21** | Smartphone | Smartphone | Smartphone | Смартфон | Smartphone | Smartphone |
| **22** | Mobile | Mobil | Móvil | Мобильный | Téléphone | Mobile |
| **23** | Portable | Tragbar | Portátil | Портативный | Portable | Portatile |
| **24** | Mobility | Mobilität | Movilidad | Мобильность | Mobilité | Mobilità |
| **25** | Flexibility | Flexibilität | Flexibilidad | Гибкость | Flexibilité | Flessibilità |
| **26** | Manoeuvrability | Manövrierfähigkeit | Maniobrabilidad | Маневренность | Manœuvre | Manovrabilità |
| **27** | Portability | Transportierbarkeit | Portabilidad | Портативность | Portabilité | Portabilità |
| **28** | Movability | Beweglichkeit | Movilidad | Подвижность | Agilité | Movibilità |
| **29** | Online | Online | Online | Онлайн | Online | Online |
| **30** | Accessible | Zugänglich | Accessible | Доступный | Accessible | Accessibile |
| **31** | Connected | Vernetzt | Conectado | Cоединенный | Connecté | Connesso |
| **32** | Virtual | Virtuell | Virtual | Bиртуальный | Virtuel | Virtuale |
| **33** | Interface | Schnittstelle | Interfaz | Интерфейс | Interface | Interfaccia |

*Note.* Digitalization word list [63] and [64] and modified by the authors of this study based on the judgements of native speakers.

**S3 Table. Finalized word set for Religion.**

|  | **British English** | **German** | **Spanish** | **Russian** | **French** | **Italian** |
| --- | --- | --- | --- | --- | --- | --- |
| **1** | Altar | Altar | Altar | Алтарь | Autel | Altare |
| **2** | Angel | Engel | Ángel | Ангел | Ange | Angelo |
| **3** | Belief | Glaube | Fe | Вера | Foi | Fede |
| **4** | Clergy | Geistlichkeit | Clero | Духовенство | Clergé | Clero |
| **5** | Creed | Überzeugung | Credo | Крид | Croyance | Credo |
| **6** | Doctrine | Doktrin | Doctrina | Доктрина | Doctrine | Dottrina |
| **7** | Heaven | Himmel | Cielo | Небеса | Ciel | Paradiso |
| **8** | Miracle | Wunder | Milagro | Чудо | Miracle | Miracolo |
| **9** | Pilgrimage | Pilgerfahrt | Peregrinación | Паломничество | Pèlerinage | Pellegrinaggio |
| **10** | Prayer | Gebet | Oración | Молитва | Prière | Preghiera |
| **11** | Prophet | Prophet | Profeta | Пророк | Prophète | Profeta |
| **12** | Religion | Religion | Religión | Религия | Religion | Religione |
| **13** | Revelation | Offenbarung | Revelación | Откровение | Révélation | Rivelazione |
| **14** | Ritual | Ritual | Ritual | Ритуал | Rituel | Rituale |
| **15** | Saint | Heiliger | Santo | Святой | Saint | Santo |
| **16** | Sermon | Predigt | Sermón | Проповедь | Sermon | Predica |
| **17** | Shrine | Schrein | Santuario | Святилище | Lieu | Santuario |
| **18** | Soul | Seele | Alma | Душа | Âme | Anima |
| **19** | Spirit | Geist | Espíritu | Дух | Esprit | Spirito |

*Note.* Religion word list [70], modified [54], and modified by the authors of this study based on the judgements of native speakers.

**S4 Table. Correlation matrix English words from 1970 till 2019.**

|  |  | | **1** | | **2** | **3** | | **4** | | **5** |
| --- | --- | --- | --- | --- | --- | --- | --- | --- | --- | --- |
|  | **1 Anxiety** | |  | |  |  | |  | |  |
|  | **2 Depression** | | .96*** | |  |  | |  | |  |
|  | **3 Digitalization** | | -.73*** | | -.77*** |  | |  | |  |
|  | **4 Years** | | .81*** | | .8*** | -.29*** | |  | |  |
| **Control** | |  | |  | | |  | |  | |
|  | **5 Religion** | | .67*** | | .76*** | -.25* | | .92*** | |  |

Note. * p<.05; ** p<.01; *** p<.001.

|  |  | | **1** | | **2** | **3** | | **4** | | **5** |
| --- | --- | --- | --- | --- | --- | --- | --- | --- | --- | --- |
|  | **1 Anxiety** | |  | |  |  | |  | |  |
|  | **2 Depression** | | .88*** | |  |  | |  | |  |
|  | **3 Digitalization** | | .56*** | | .51*** |  | |  | |  |
|  | **4 Years** | | .88*** | | .6*** | .65*** | |  | |  |
| **Control** | |  | |  | | |  | |  | |
|  | **5 Religion** | | -.45*** | | -.62*** | -.63*** | | -.34** | |  |

**S5 Table. Correlation matrix German words from 1970 till 2019.**

Note. * p<.05; ** p<.01; *** p<.001.

|  |  | | **1** | | **2** | **3** | | **4** | | **5** |
| --- | --- | --- | --- | --- | --- | --- | --- | --- | --- | --- |
|  | **1 Anxiety** | |  | |  |  | |  | |  |
|  | **2 Depression** | | .94*** | |  |  | |  | |  |
|  | **3 Digitalization** | | .91*** | | .87*** |  | |  | |  |
|  | **4 Years** | | .82*** | | .7*** | .95*** | |  | |  |
| **Control** | |  | |  | | |  | |  | |
|  | **5 Religion** | | -.75*** | | -.6*** | -.89*** | | -.95*** | |  |

**S6 Table. Correlation matrix Spanish words from 1970 till 2019.**

Note. * p<.05; ** p<.01; *** p<.001.

**S7 Table. Correlation matrix Russian words from 1970 till 2019.**

|  |  | | 1 | | 2 | 3 | | 4 | | 5 |
| --- | --- | --- | --- | --- | --- | --- | --- | --- | --- | --- |
|  | **1 Anxiety** | |  | |  |  | |  | |  |
|  | **2 Depression** | | .99*** | |  |  | |  | |  |
|  | **3 Digitalization** | | -.34*** | | -.39*** |  | |  | |  |
|  | **4 Years** | | .93*** | | .93*** | -.33*** | |  | |  |
| **Control** | |  | |  | | |  | |  | |
|  | **5 Religion** | | .81*** | | .84*** | -.69*** | | .87*** | |  |

Note. * p<.05; ** p<.01; *** p<.001.

|  |  | | **1** | | **2** | **3** | | **4** | | **5** |
| --- | --- | --- | --- | --- | --- | --- | --- | --- | --- | --- |
|  | **1 Anxiety** | |  | |  |  | |  | |  |
|  | **2 Depression** | | .98*** | |  |  | |  | |  |
|  | **3 Digitalization** | | .79*** | | .75*** |  | |  | |  |
|  | **4 Years** | | .93*** | | .84*** | .72*** | |  | |  |
| **Control** | |  | |  | | |  | |  | |
|  | **5 Religion** | | -.74*** | | -.81*** | -.74*** | | -.48*** | |  |

**S8 Table. Correlation matrix French words from 1970 till 2019.**

Note. * p<.05; ** p<.01; *** p<.001.

**S9 Table. Correlation matrix Italian words from 1970 till 2019.**

|  |  | | **1** | | **2** | **3** | | **4** | | **5** |
| --- | --- | --- | --- | --- | --- | --- | --- | --- | --- | --- |
|  | **1 Anxiety** | |  | |  |  | |  | |  |
|  | **2 Depression** | | .9*** | |  |  | |  | |  |
|  | **3 Digitalization** | | .66*** | | .43*** |  | |  | |  |
|  | **4 Years** | | .62*** | | .32*** | .94*** | |  | |  |
| **Control** | |  | |  | | |  | |  | |
|  | **5 Religion** | | -.84*** | | -.78*** | -.38*** | | -.28* | |  |

Note. * p<.05; ** p<.01; *** p<.001.

**S10 Table. Correlation matrix English Fiction words from 1970 till 2019.**

Note. * p<.05; ** p<.01; *** p<.001.

|  |  | | **1** | | **2** | **3** | | **4** | | **5** |
| --- | --- | --- | --- | --- | --- | --- | --- | --- | --- | --- |
|  | **1 Anxiety** | |  | |  |  | |  | |  |
|  | **2 Depression** | | .64*** | |  |  | |  | |  |
|  | **3 Digitalization** | | .16 | | .71*** |  | |  | |  |
|  | **4 Years** | | -.32** | | -.9*** | -.83*** | |  | |  |
| **Control** | |  | |  | | |  | |  | |
|  | **5 Religion** | | .07 | | .77*** | .72*** | | -.85*** | |  |


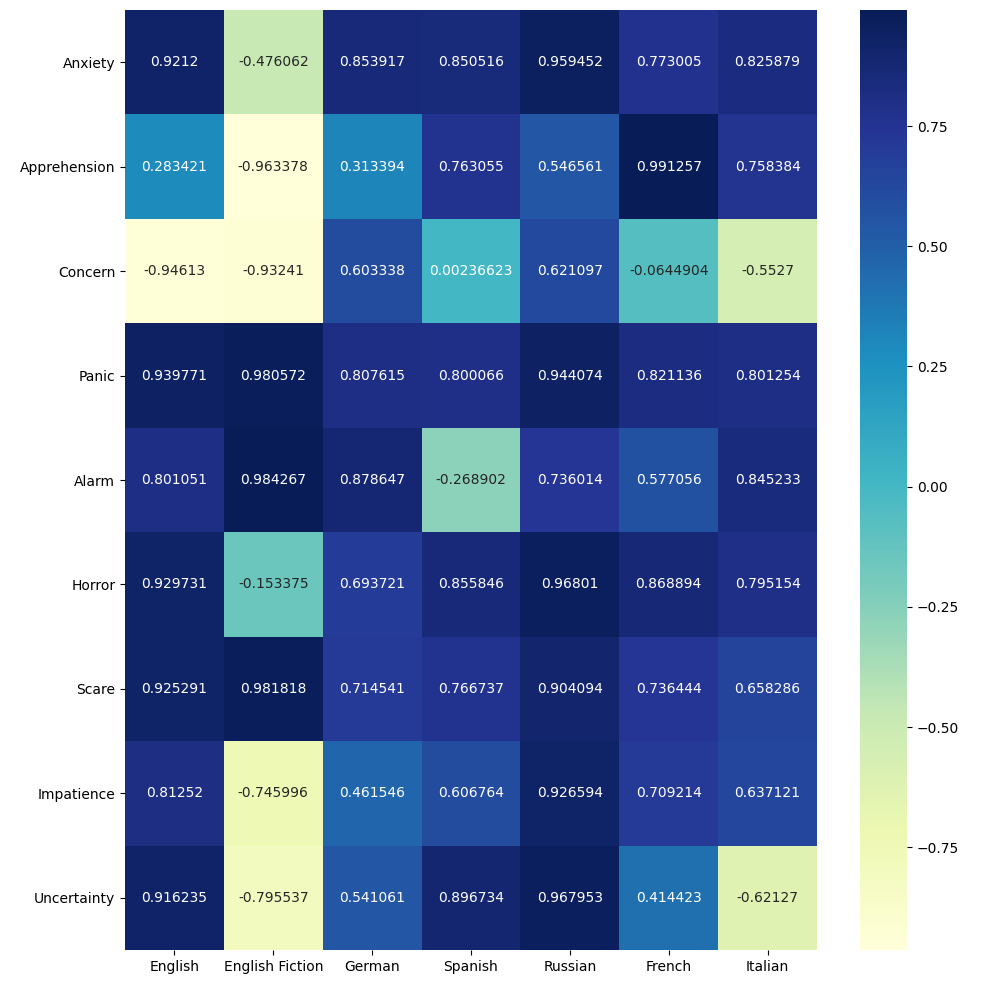


**S1 Fig. Visual representation of correlation coefficients for each anxiety word correlated with years.**


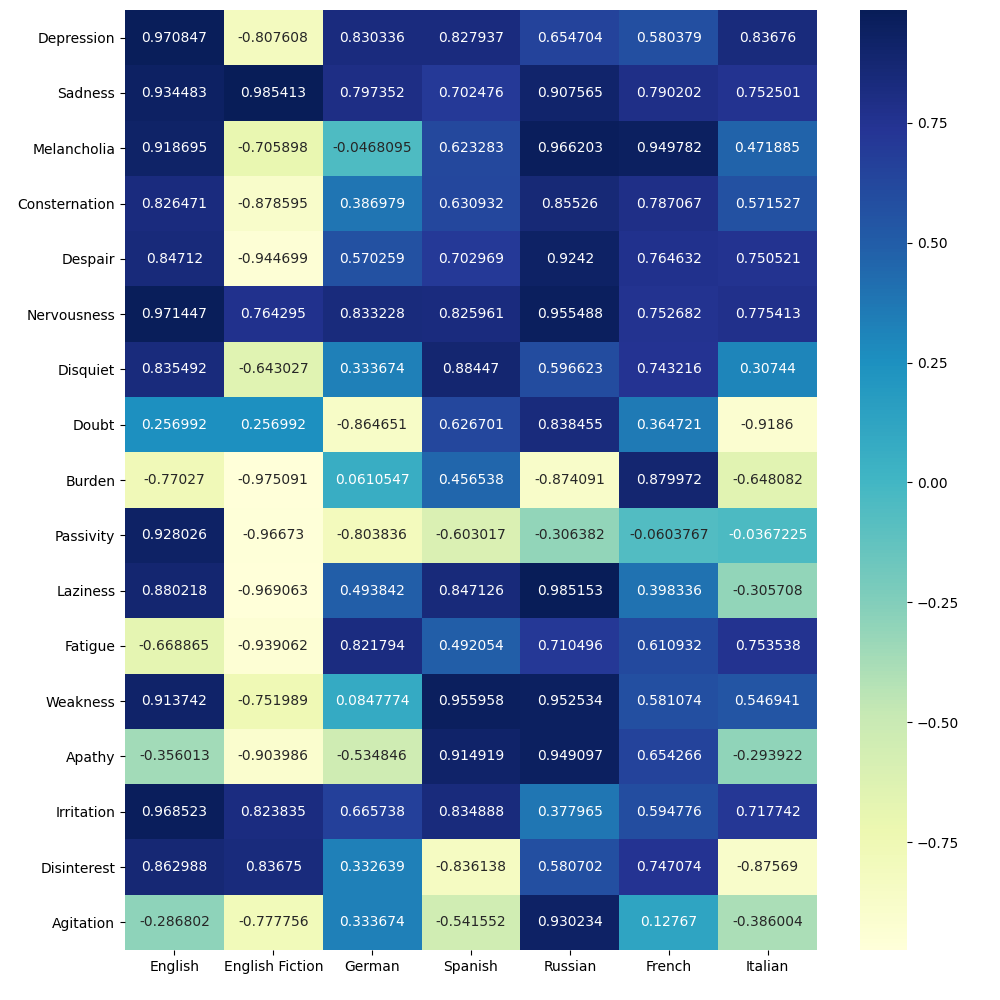


**S2 Fig. Visual representation of correlation coefficients for each depression word correlated with years.**


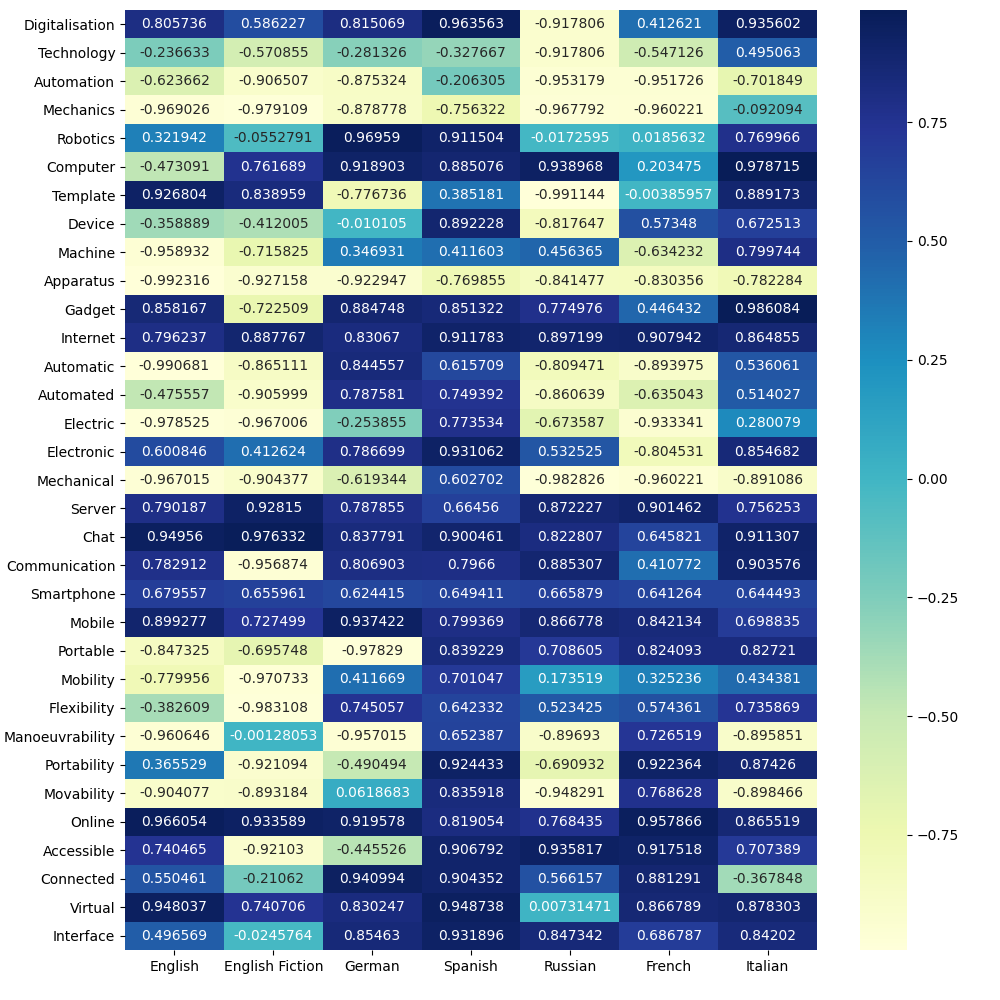


**S3 Fig. Visual representation of correlation coefficients for each digitalization word correlated with years.**


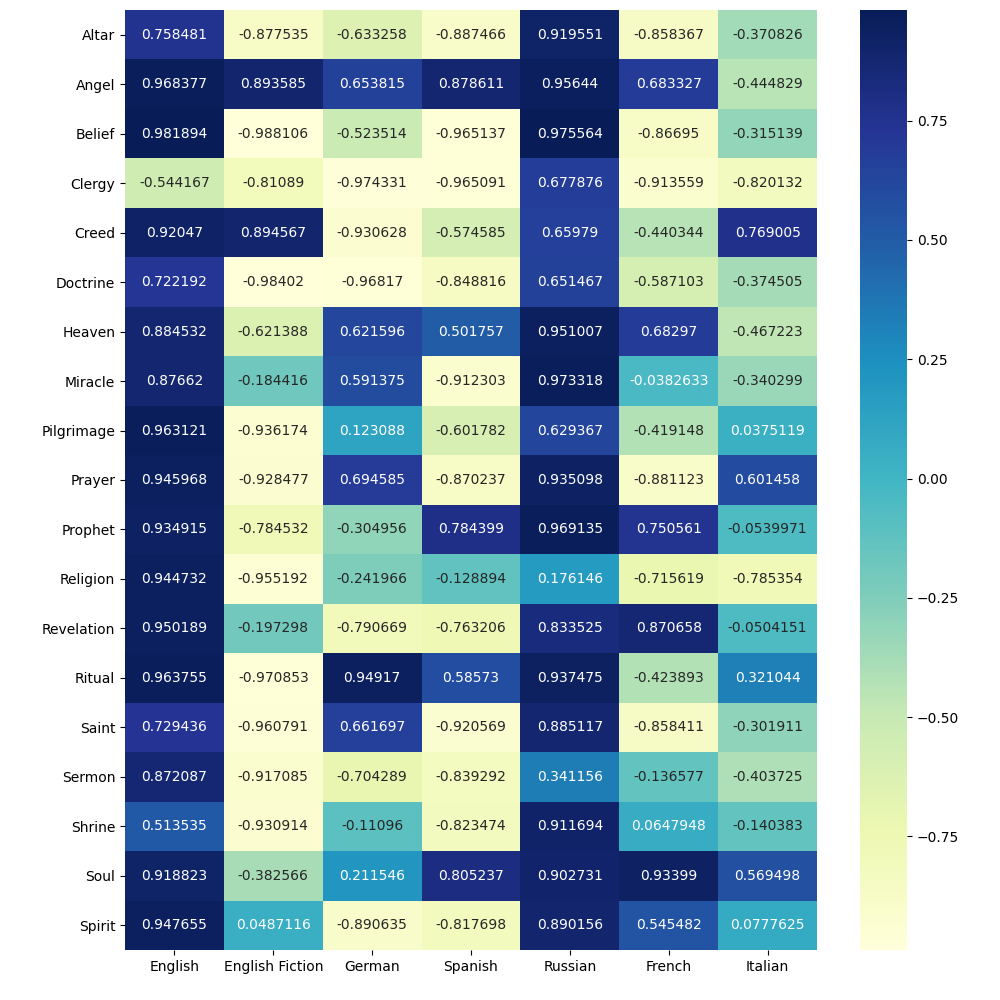


**S4 Fig. Visual representation of correlation coefficients for each religion word correlated with years.**


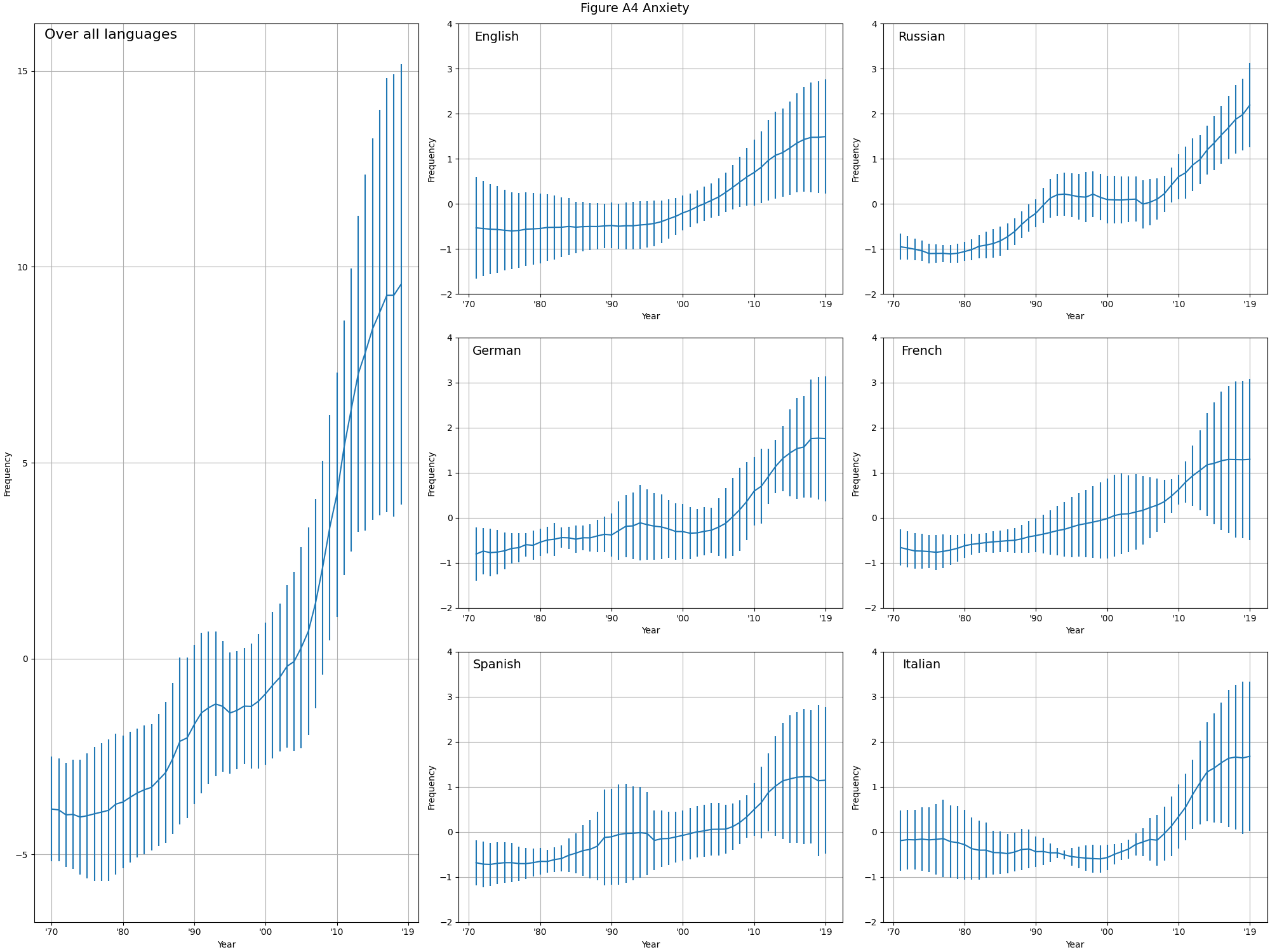


**S5 Fig. Mean and standard deviation of frequency for all anxiety words over time with standard deviation.**


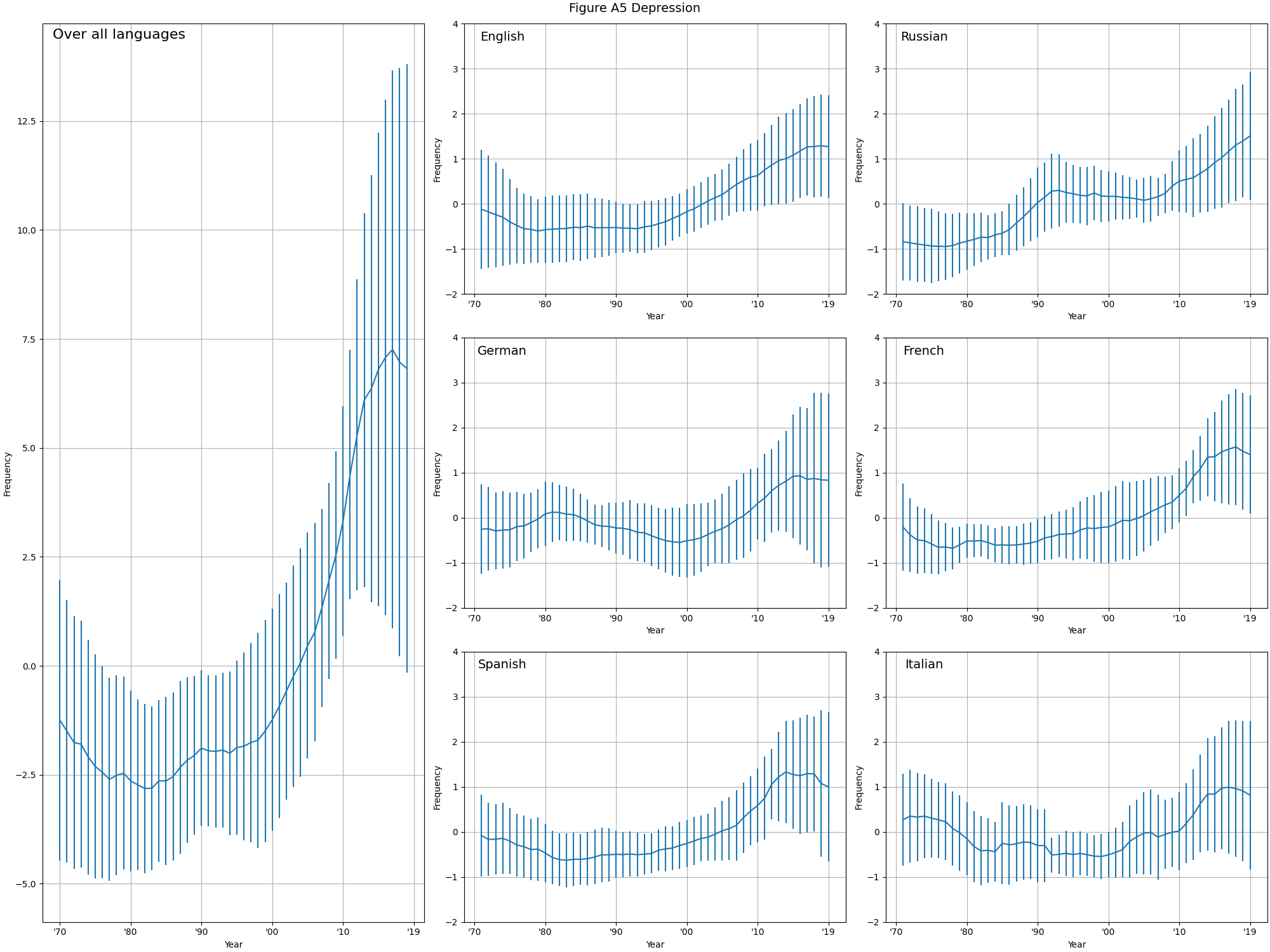


**S6 Fig. Mean and standard deviation of frequency for all depression words over time with standard deviation.**


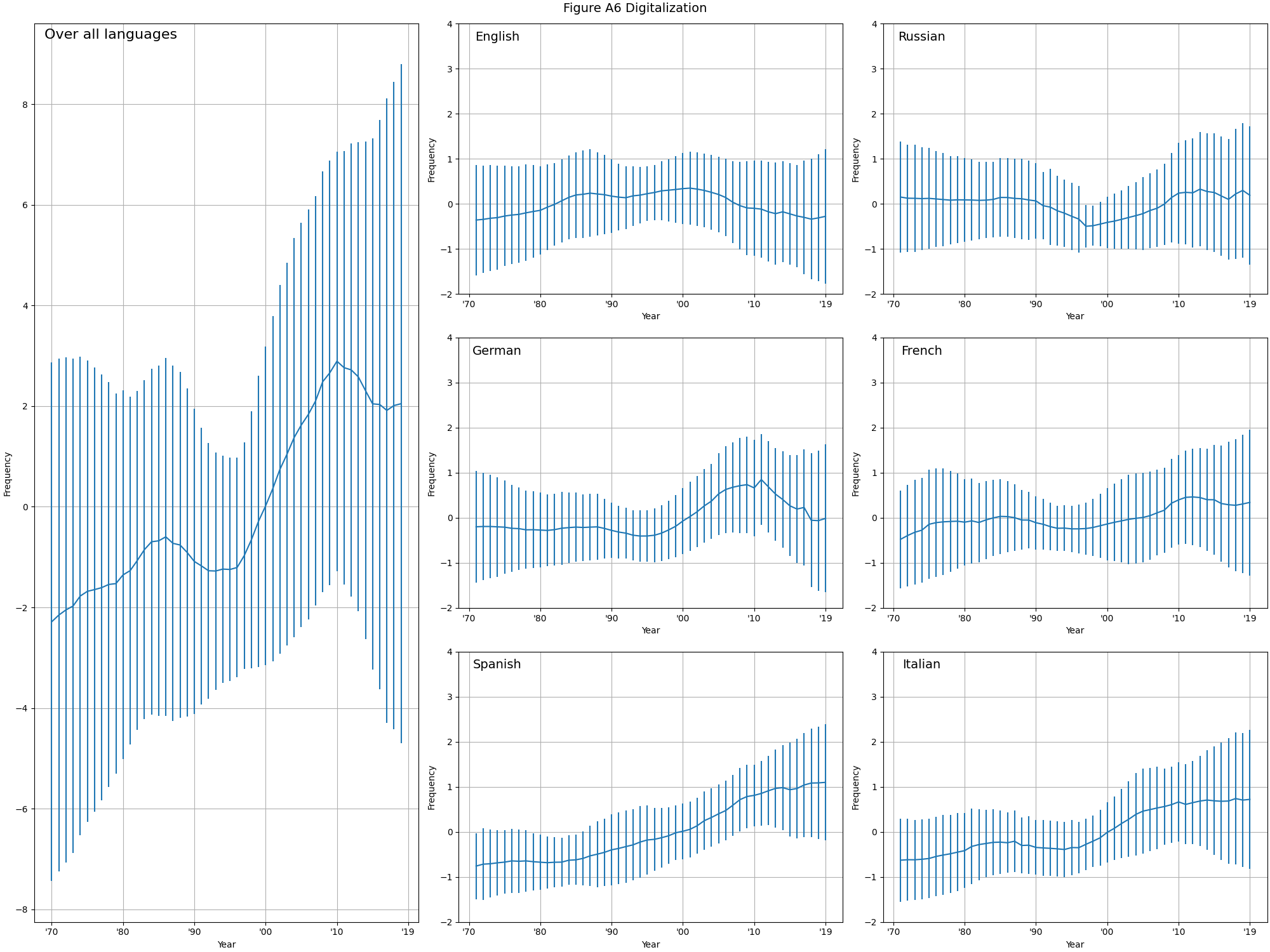


**S7 Fig. Mean and standard deviation of frequency for all digitalization words over time with standard deviation.**


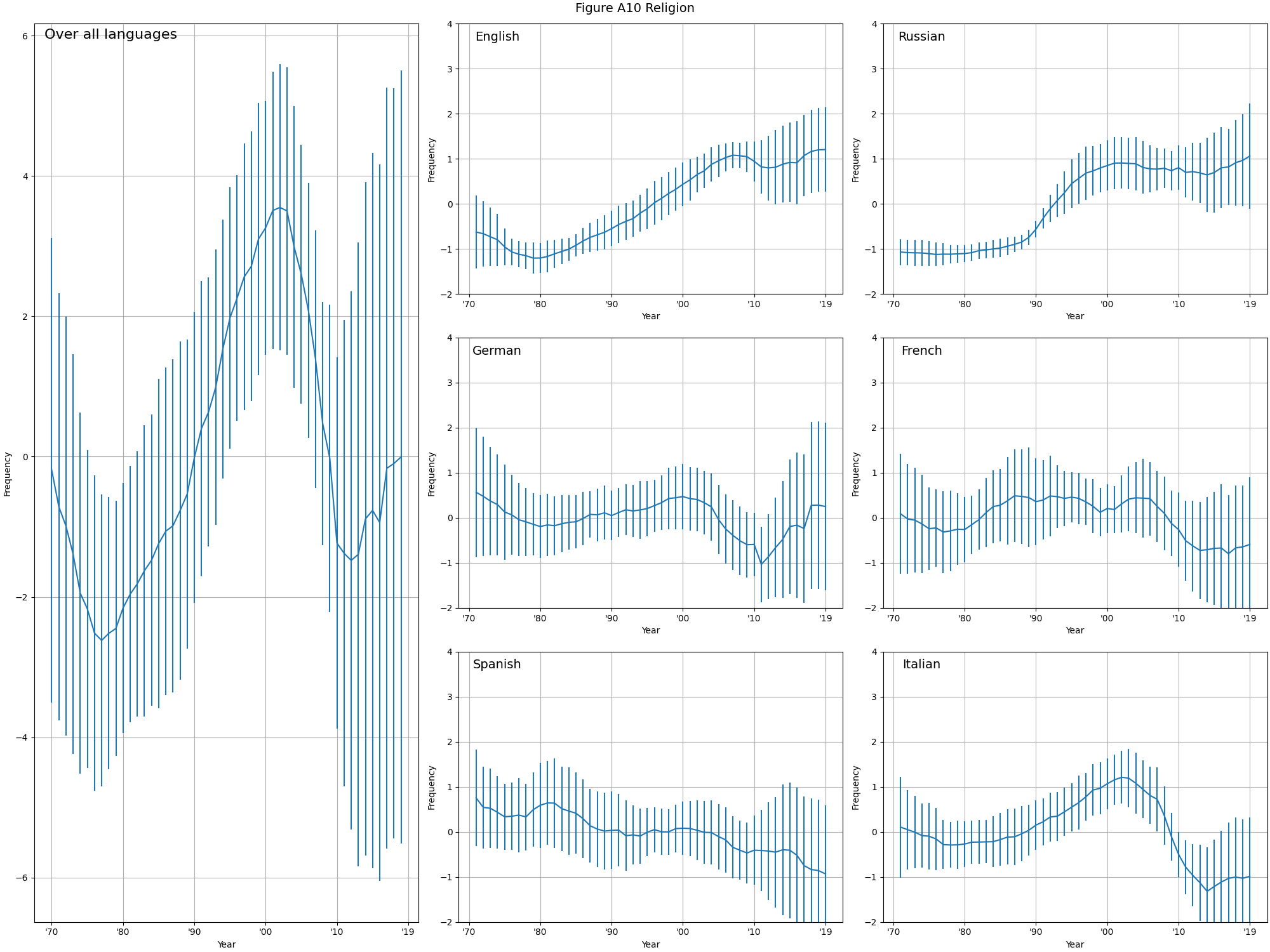


**S8 Fig. Mean and standard deviation of frequency for all religion words over time with standard deviation.**


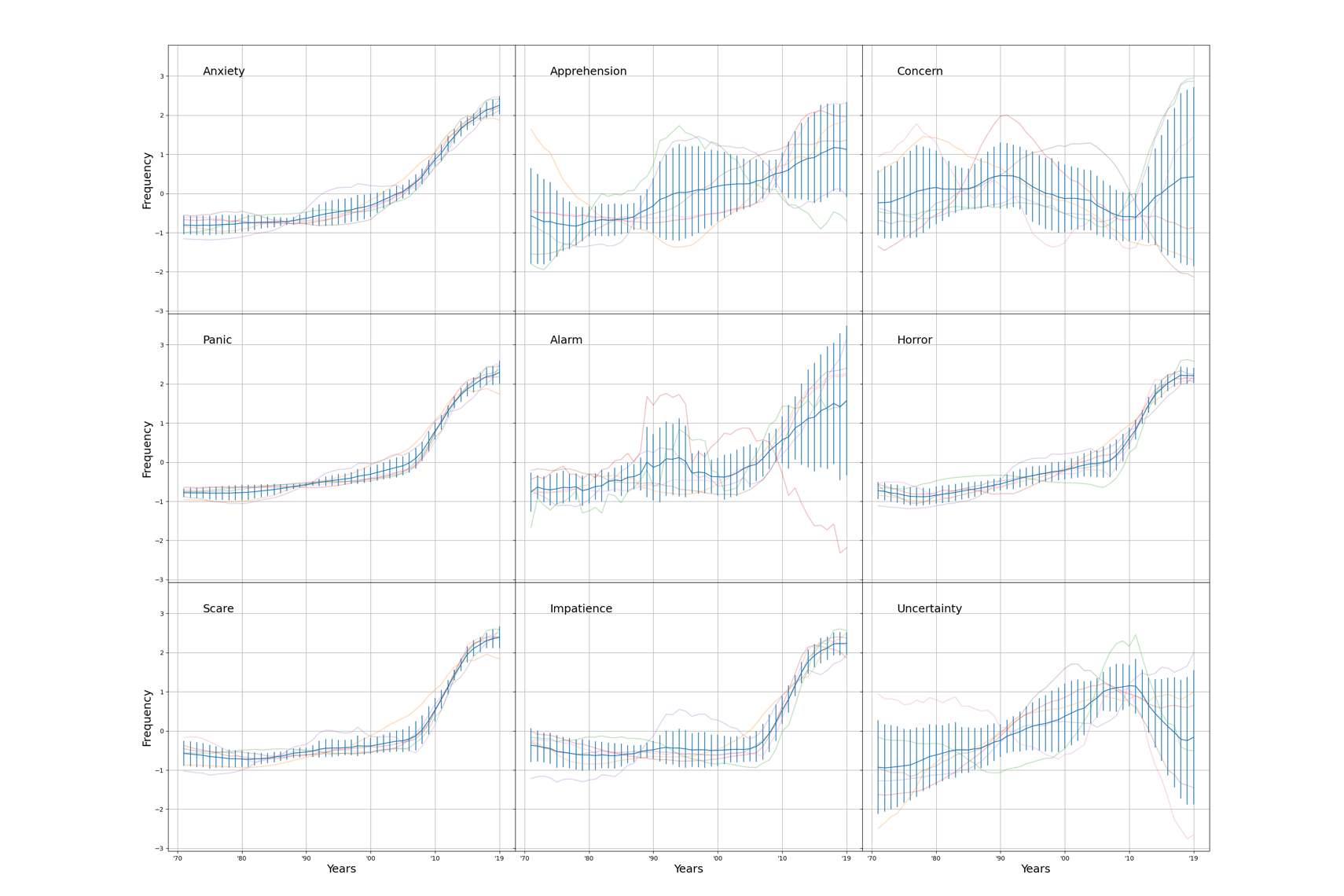


**S9 Fig. Frequency change for each anxiety word for each language and averaged over all languages.** Displayed as blue line.


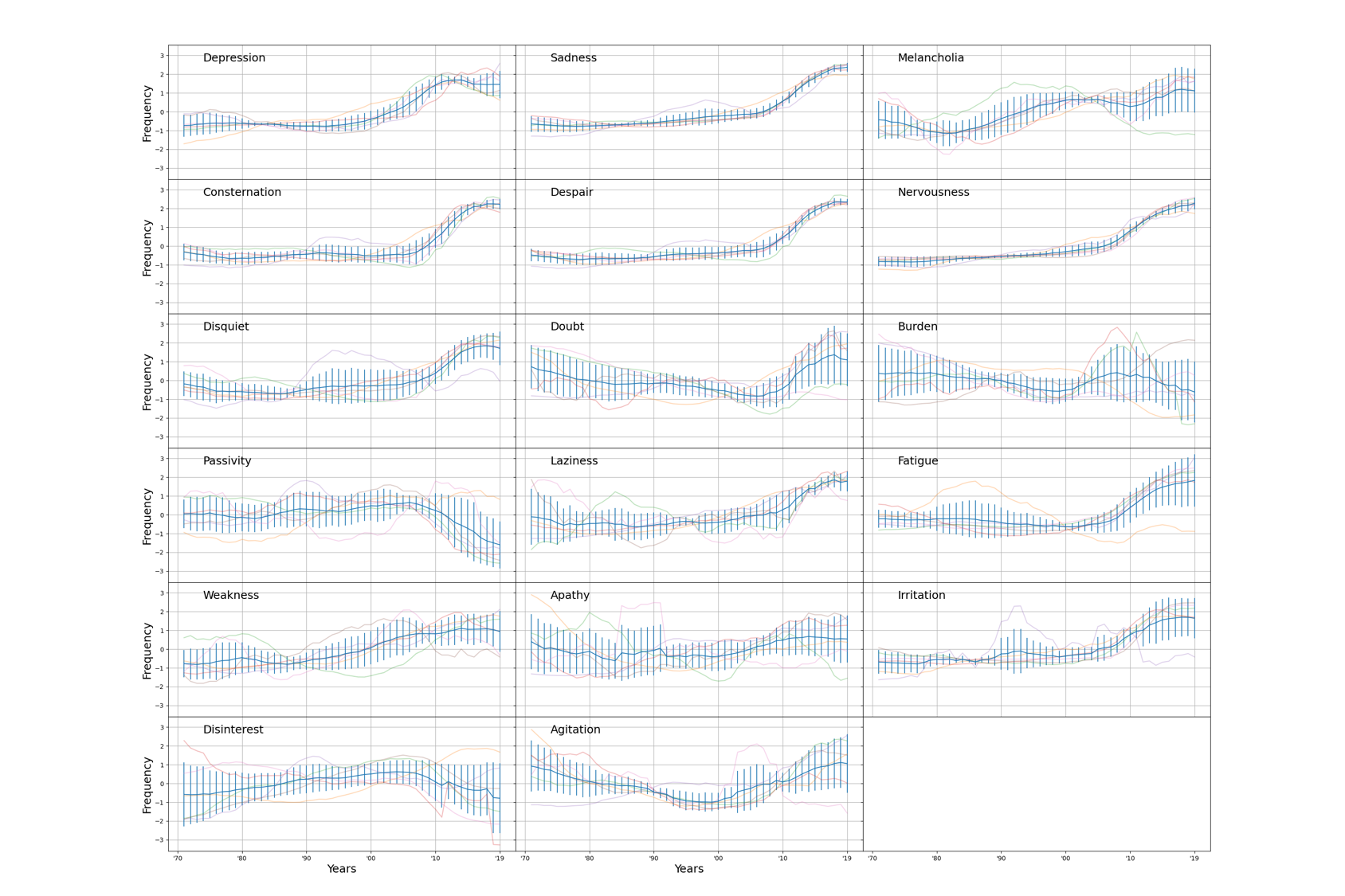


**S10 Fig. Frequency change for each depression word for each language and averaged over all languages.** Displayed as blue line.


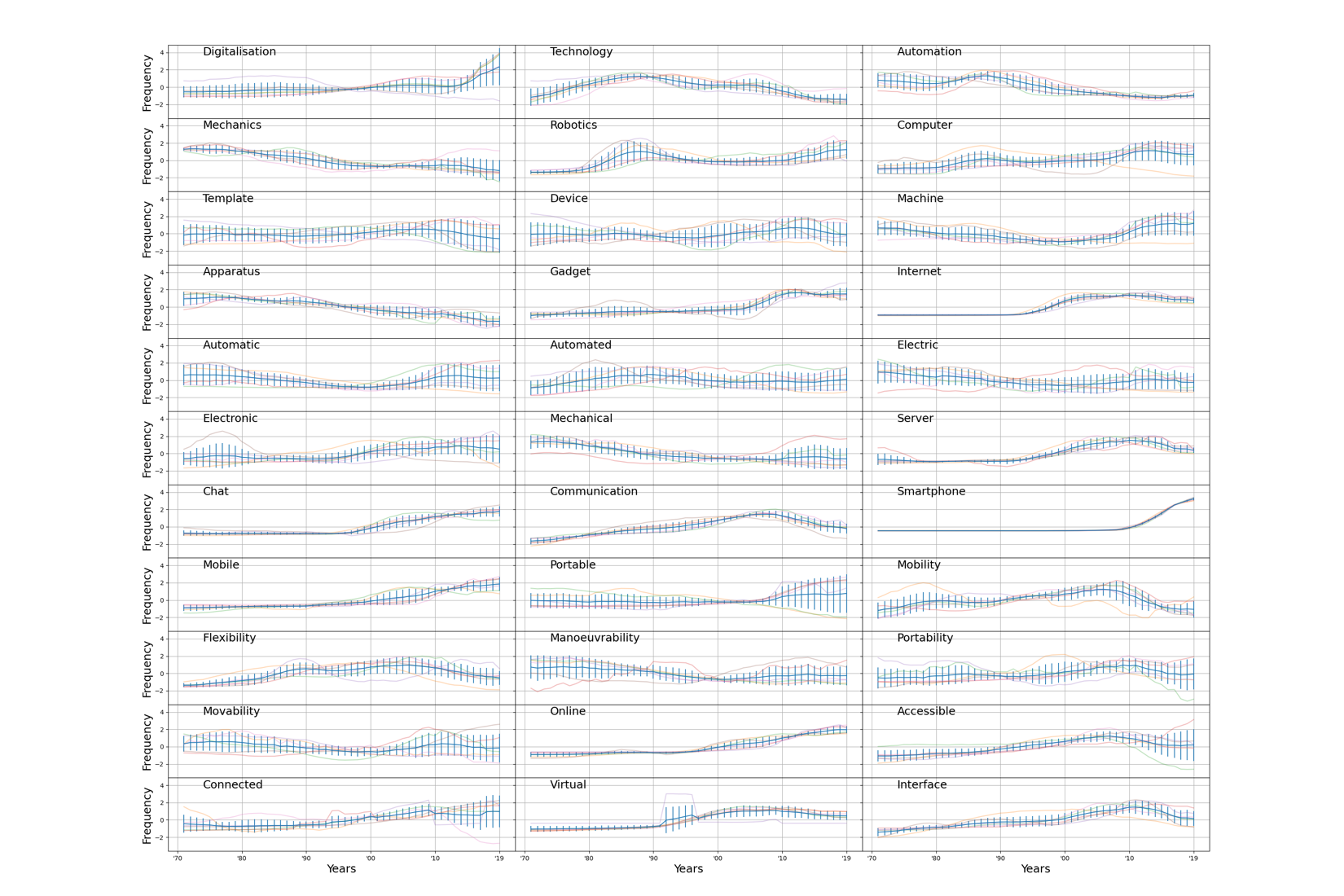


**S11 Fig. Frequency change for each digitalization word and averaged over all languages.** Displayed as blue line.


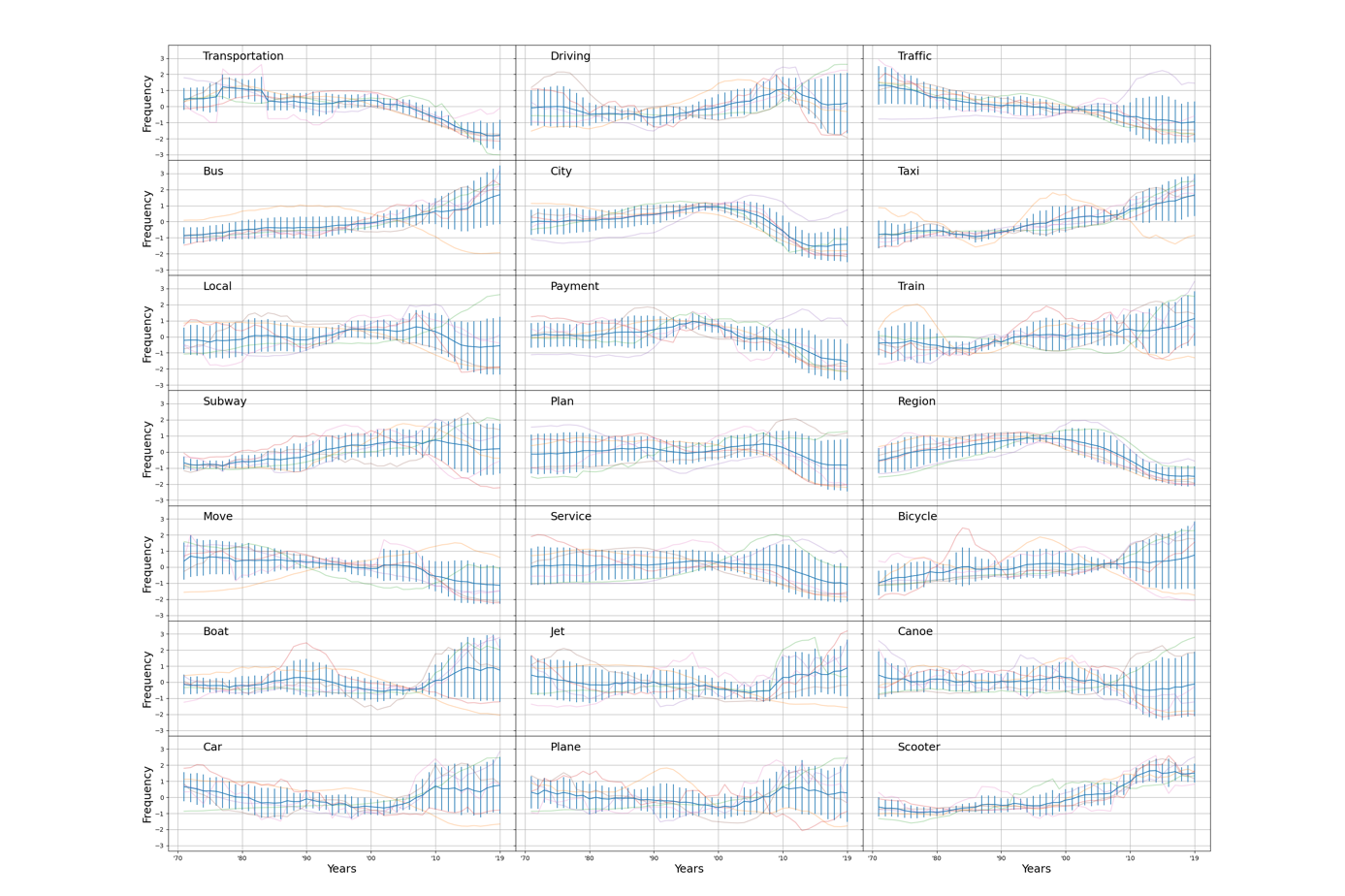


**S12 Fig. Frequency change for each digitalization word averaged over all languages.** Displayed as blue line.


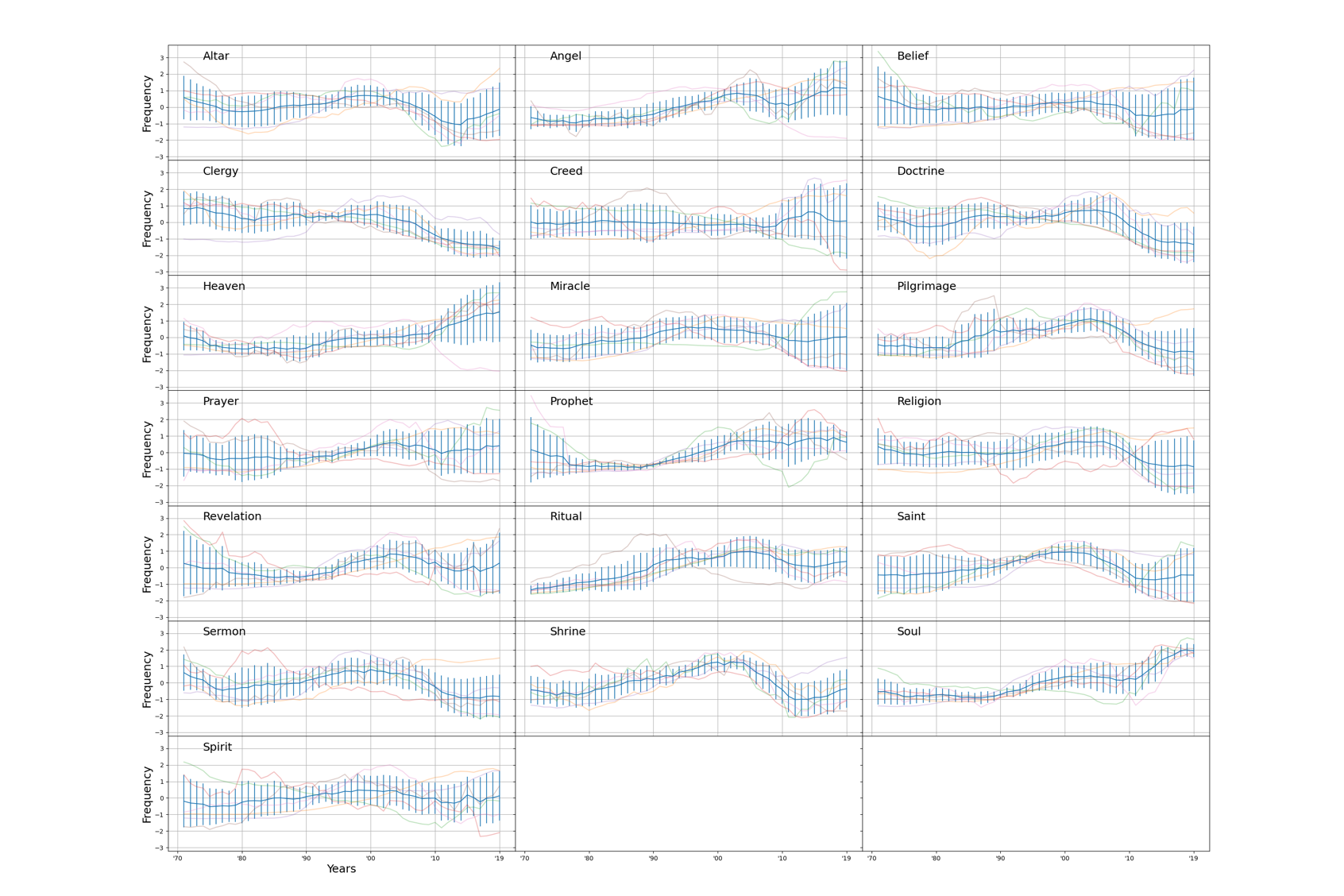


**S13 Fig. Frequency change for each digitalization word averaged over all languages.** Displayed as blue line.


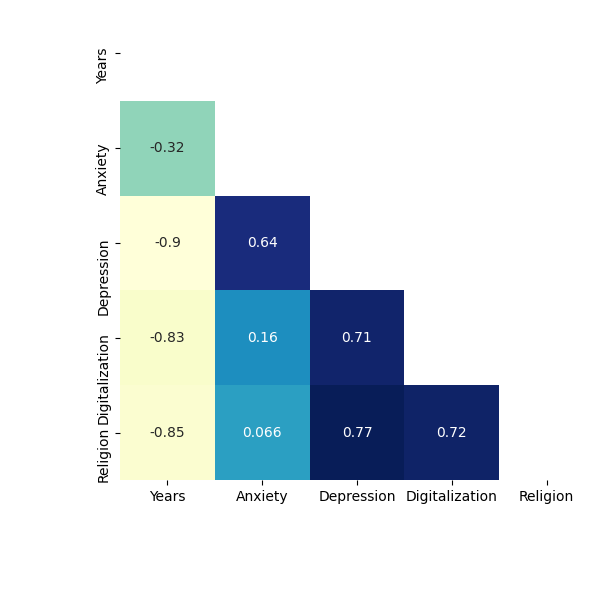


**S14 Fig. Correlation coefficients for English Fiction words by word category.**
